# Subtitling for d/Deaf and Hard-of-Hearing Children: Current Practices and New Possibilities to Enhance Language Development

**DOI:** 10.3390/brainsci7070075

**Published:** 2017-06-30

**Authors:** Ana Tamayo, Frederic Chaume

**Affiliations:** 1 TRALIMA/ITZULIK Consolidated Research Group GIU 16/48, Department of English and German Philology and Translation and Interpreting, Universidad del País Vasco UPV/EHU, Vitoria-Gasteiz E-01006, Spain; 2 TRAMA Research Group, Universitat Jaume I, Department of Translation and Communication, Castelló de la Plana E-12071, Spain; frederic.chaume@trad.uji.es

**Keywords:** subtitling, SDH, captioning, audiovisual translation, accessibility, deaf children, linguistic code, vocabulary, syntax

## Abstract

In order to understand and fully comprehend a subtitle, two parameters within the linguistic code of audiovisual texts are key in the processing of the subtitle itself, namely, vocabulary and syntax. Through a descriptive and experimental study, the present article explores the transfer of the linguistic code of audiovisual texts in subtitling for deaf and hard-of-hearing children in three Spanish TV stations. In the first part of the study, we examine current practices in Spanish TV captioning to analyse whether syntax and vocabulary are adapted to satisfy deaf children’s needs and expectations regarding subtitle processing. In the second part, we propose some alternative captioning criteria for these two variables based on the needs of d/Deaf and hard-of-hearing (DHH) children, suggesting a more appropriate way of displaying the written linguistic code for deaf children. Although no specific distinction will be made throughout this paper, it is important to refer to these terms as they have been widely used in the literature. Neves (2008) distinguishes between the “Deaf”, who belong to a linguistic minority, use sign language as their mother tongue, and usually identify with a Deaf community and culture; the “deaf”, who normally have an oral language as their mother tongue and feel part of the hearing community; and the “hard of hearing”, who have residual hearing and, therefore, share the world and the sound experience of hearers. In the experimental study, 75 Spanish DHH children aged between 8 and 13 were exposed to two options: the actual broadcast captions on TV, and the alternative captions created by the authors. The data gathered from this exposure were used to analyse the children’s comprehension of these two variables in order to draw conclusions about the suitability of the changes proposed in the alternative subtitles.

## 1. Introduction

There is little doubt that, nowadays, audiovisual products lead the way we access and consume culture and information from an early age [1], and as such, are essential to how oral and written language is acquired and developed. Audiovisual products are multisemiotic constructs that convey information through auditory and visual channels in the form of verbal (e.g., dialogues) and non-verbal signs (e.g., facial expressions), and codes [2,3,4]. Spoken (and written) language—the linguistic code—is therefore only one of the many signifying codes [2,3] in audiovisual products, and interacts with other signs and codes to create audiovisual meaning. These other signs and codes include paralinguistic features of the voice, sounds, music, gestures, film shots, lighting, etc. Although it is only one of the many codes that create meaning, the linguistic code is usually the most essential to understanding audiovisual material, and will ultimately have major implications in the language development of any audience.

Spoken language in audiovisual products has traditionally been conveyed in two ways for DHH audiences: sign language interpreting (SLI) and subtitling for the DHH (SDH). Of these two, SDH seems to have gained importance in the audiovisual translation field, essentially for two reasons. First, it is a relatively cheap and fast solution, and second, the DHH community is moving towards orality as most DHH children, at least in Spain, are being educated in mainstream schools [5] and 89.6% of children and teenagers in deaf people’s organisations communicate through an oral language [6], which makes sense given that 90% of DHH children are born to hearing parents [7]. Additionally, 90% of children and teenagers use oral language at school and, in a study with 586 families, the Spanish Confederation of Families of Deaf People (FIAPAS) found that all children and teenagers in deaf people’s organisations use some kind of hearing device [6]. As a result, although sign language interpreting is still important, it is gradually becoming less relevant for the DHH community as a whole. In subtitling for the DHH, spoken language is conveyed through subtitles, which become part of the multisemiotic construct of the audiovisual product.

DHH audiences, whether their mother tongue is an oral language or a sign language, encounter difficulties in understanding the written linguistic code both in static text and in captioned audiovisual products. There is still much debate on whether DHH people who use a signed or oral language as their L1 experience difficulties in interpreting print language for example, [8], although most research finds that obstacles are mainly due to two variables—vocabulary and syntax cf. [9,10,11,12,13,14,15,16,17] among others.

In fact, “deaf children starting school at the age of four of five have, on average, 500 words as part of their vocabulary as opposed to the 3000–5000 words known by hearing children” [17,18] and find it more difficult to acquire figurative language [17,19]. New vocabulary is one of greatest hurdles to reading comprehension [15]. In the late 70s, Gormley and Franzen [4,20] found that “the deaf, particularly good deaf readers, may bypass the surface structure of syntax and process written information at the deep structure level of semantic information”. Additionally, “linear structures are easier to comprehend than hierarchical ones” [17,21], and deaf children may therefore find it more difficult to process complex syntactic structures such as relative clauses [17,22]. Most of the research mentioned examines either oral or static print language, not the two simultaneously within audiovisual content. In this regard, the subtitler’s task is far from straightforward, as the extent to which their target audience is able to recognise and decode new vocabulary or complex syntax within the audiovisual text is not immediately obvious.

Although these variables must be taken into account when subtitling for any audience, regardless of their access to sound, they have a far more determining effect on the DHH audience’s comprehension of subtitles, which can be limited by both short-term memory and DHH community heterogeneity [14,23]. Furthermore, DHH children “lag considerably behind hearing learners in their reading achievements; have limited vocabulary acquisition and knowledge of multiple meanings; have poor knowledge of semantics and syntax, and a delayed knowledge of grammatical rules” [24,25,26]. Moreover, vocabulary and syntax do not operate separately [11], rather “the relationship between vocabulary and reading comprehension is dependent on syntactic abilities” [11,26]. However, the implications these variables have for language acquisition are not the same. Some studies suggest that vocabulary tends to improve with habitual consumption of subtitled audiovisual material cf. [12], but syntax is still one of the biggest challenges to the DHH community’s comprehension of written text and, therefore, subtitles and captioned audiovisual products.

In addition, it has long been known that language is acquired incidentally [27], that is, by exposure and focusing on content rather than on grammar [26,27]. Moreover, subtitles are not a source of distraction when watching audiovisual material [28]. As early as the 1980s and 1990s, authors like Parlato [29], Ellsworth [30], Rees [31], and Huang and Eskey [32], were highlighting the benefits of subtitling and SDH to enhance language acquisition at many levels. In Spain, Talaván [33,34] among others and Díaz-Cintas [35] have also addressed this issue. Thus, when conveyed properly, SDH can be a powerful tool to minimise the “affective filter” [27] in reading for DHH children, this filter being the emotional variables such as anxiety or stress that can affect language acquisition. Adapting vocabulary and syntax in subtitle processing might reduce the cognitive effort viewers must make to understand the text they are reading, thus allowing them to focus on watching, processing, and enjoying images—the audiovisual product as a whole, in other words—and at the same time facilitating language acquisition and development. Moreover, Silvestre and Laborda [36] argue that students with prelingual deafness (i.e., a child with profound hearing loss before acquiring language) do not acquire new vocabulary autonomously; rather, an adult explains the meaning to them, and because they are exposed to fewer inputs, deaf students have fewer opportunities to extend their use of new words. Consequently, exposure to a type of subtitling that enhances the acquisition of new vocabulary and syntax, while at the same time letting the audience enjoy the audiovisual content, might be an effective way of increasing input and promoting a more incidental and natural way of learning vocabulary, something that does not seem to occur among deaf children [37].

Deaf children are not a homogeneous group, however. They differ greatly in their active vocabulary (recalled and used when the communicative situation requires it) and passive vocabulary (understood and contextualised) [38], as well as in their knowledge of the world and syntactic structures [39]. These differences are, logically, reflected in their reading performance [40], and might be due to factors such as age, type of school they attend, hearing devices, communication method (oral or signed), degree of hearing loss, and reading habits, among others. As a result, many researchers in the field of audiovisual translation (AVT) advocate using different sets of criteria to create SDH depending on the needs and reading abilities of the audience [14,41,42,43,44] among others. This practice has yet to be extended to SDH for children however, and is unlikely to be implemented in the near future [43]. In other words, it is obvious that a postlingually hard-of-hearing adult (i.e., a person who suffered hearing loss after acquiring language) has different needs and reading abilities from a prelingually Deaf child. Similarly, an 8-year-old DHH child will probably have different reading abilities and, thus, different needs from a 13-year-old. However, despite its relatively low costs, subtitling audiovisual material implies an expense for the industry. Spain only recently regulated the amount of subtitled audiovisual material television companies must provide through the General Law of Audiovisual Communication of 2010, and we are therefore unlikely to see different types of subtitles tailored to different needs, let alone different types of subtitles for different subgroups of DHH children. Accordingly, researchers in the field have suggested simplifying the linguistic code so it can be understood by the whole DHH community, regardless of their reading abilities, communication method, hearing devices, age, or degree of hearing loss. It is worth mentioning that both the industry and deaf people’s organisations advocate literal or verbatim captioning [45,46,47,48]. Clearly, verbatim captioning can be done more quickly and is the preferred option of the industry, which in Spain is now obliged to provide high percentages of subtitled material on TV with the corresponding extra costs. On the other hand, the feeling among the DHH community, who have been traditionally left out of the information society and who for many years have had little or no access to information and communication technologies, may be that edited captioning might lack information that is accessible to hearers [47]. Their subjective preferences, however, cannot be the only criterion for deciding whether to provide literal or edited subtitling; this decision must also take into account objective data on comprehension and enjoyment of audiovisual products, among other factors.

Various recommendations have been made in the literature. Concerning vocabulary, Lorenzo [38] advocates simplifying linguistic variation, Lorenzo and Pereira [43] advise avoiding metaphors and using common vocabulary, and Neves [14] prioritises simple vocabulary, advising the use of difficult vocabulary only for a specific purpose and when viewers have enough time to process its meaning. Zárate [17] also recommends enhancing new or difficult vocabulary (with a different font type or size), reducing the subtitle speed, which is in line with recent guidelines cf. [49], and interphrasal redundancy, to help acquisition of new words [26,50].

With regard to syntax, “deaf viewers will benefit from subtitles that are syntactically and semantically structured in ways that will facilitate reading. Long complex sentences will obviously be more demanding on their short-term working memory” [14]. Here, Neves [14] is advocating short, direct structures with logical subtitle segmentation; Lorenzo and Pereira [43] also recommend simple syntactic structures and short sentences.

Despite discrepancies over whether DHH children with access to a natural language (signed or oral) have difficulties in interpreting print language, and about the best ways to convey audible linguistic code into subtitles (verbatim or edited), this study follows recent calls from AVT scholars for changes that will promote the use of alternative subtitles. We test these alternative subtitles against currently available SDH in an attempt discover whether edited subtitles can facilitate subtitle comprehension.

## 2. Methods and Materials

This research presents two clearly differentiated but complementary studies describing current practices in the captioning of the linguistic code, both concerned with vocabulary and syntax, and experimenting with alternative solutions based on previous research and existing technical resources to enhance language acquisition and development.

The first part is a descriptive study of the current practices of Spain’s three digital terrestrial television (DTTV) stations dedicated to young audiences (aged approximately between 4 and 12), namely, *Boing*, *Clan*, and *Disney Channel*. Data were gathered by recording audiovisual material directly from the DTTV stations with the Easy Home Combo HD DVD player on USB memory in .ts files, which were then opened with VLC (VideoLAN media player), a free open-source cross-platform multimedia player, to analyse the linguistic code of the subtitles in a visual and acoustic context.

This preliminary descriptive study of the first variable, vocabulary, analysed whether difficult vocabulary was present in the spoken language. The thorny question of “What is ”difficult vocabulary”?” was addressed following recommendations in previous research cf. [14,38,43] and by restricting the variable to technical terms, idiomatic expressions, and colloquial language. When difficult vocabulary was found in the acoustic channel, a decision was made either to alter it (omit, adapt, or simplify to facilitate comprehension), or to convey it verbatim into subtitles. Analysis of the second variable, syntax, focused on whether subtitles followed a simple and canonical syntactic structure (subject-verb-object), and whether subordinate clauses were used. Data on both variables were imported into IBM SPSS Statistics software (Cary, NC, USA). Thus, although the first part of the study entails a qualitative discussion of current practices, quantitative data were taken into account in the qualitative analysis.

A corpus relevant to the present study was selected following methodologically reasoned criteria taken from descriptive translation studies (compiling a coherent catalogue of audiovisual texts, filtering the catalogue into a representative and manageable corpus, etc.). These criteria are explained in detail in the literature cf. [44,51,52,53].

The second part of the research is an experimental study comparing DHH children’s comprehension of the linguistic code when they are exposed to current practices (verbatim transcription of spoken language into subtitles without the use of orthotypographic resources to enhance language acquisition) with exposure to an alternative set of subtitles created by the authors. To create the new alternative subtitles, audiovisual products recorded with Easy Home Combo HD were converted from .ts to .mpg2 files using the Xilisoft Video Converter and then cut into smaller audiovisual units with Smart Cutter. The videos were then uploaded onto Aegisub subtitling software to create SDH in .ass format. The subtitle files were given the same label as the audiovisual files to ensure synchronised and automatic viewing with VLC.

The alternative captions were created by making the changes in the variables, as shown in Table 1, taking into account recommendations from the authors mentioned above:

Changes in vocabulary and syntax were made, when applicable, in all subtitles. Modifying both variables in the alternative subtitles instead of isolating the changes made it difficult to determine the effects of the changes individually. Nevertheless, as pointed out in previous research [41] and taking into consideration the dependency of vocabulary on syntactic abilities noted above, just changing isolated parameters does not account for the interdependency of all parameters in creating SDH.

A total of 75 DHH children (34 boys and 41 girls), aged between 8 and 13 ($\bar{x}$ = 10.32) took part in the experiments. In all cases, parental or tutor consent was signed before participants took part in the experiment, which was also approved by the Ethics Committee (*Comisión Deontológica*) at Universitat Jaume I (Castellón, Spain) on 11 February 2015. Given that the Spanish UNE Standard 153010 [54] does not consider the heterogeneous needs of the DHH community, subjects were selected from a wide age range. This decision was made so as to test the alternative subtitles against the current practices in a heterogeneous group of DHH children, also bearing in mind that the industry would not be willing to tailor subtitling types to DHH children’s different needs. All participants viewed the same number of video files (14) either with the actual TV captioning (TV) or with the alternative captioning (ALT). All subjects were exposed to the videos with sound in order to achieve the most natural viewing atmosphere possible, taking into account that most subjects had either hearing aids or cochlear implants (73 out of 75 participants), and had the oral language as their only mother tongue, or were bilingual in sign and oral language (only 3 participants stated that sign language was their only natural communication method). For the purposes of this study, the following selection criteria were established:

- Aged between 8 and 13 years old
- Resident in Spain
- DHH and with no other associated impairment
- Mother tongue either oral Spanish or any natural sign language. If a sign language was the mother tongue, oral Spanish was required as a second language. This decision was taken to minimise the effect of having to deal with subtitles in a foreign language (L3).
- At least occasional use of captioning on TV

No probabilistic techniques were used to select participants for various reasons. First, we wanted to reach as many participants as possible, and second, in Spain we do not know the exact population of young DHH audiences cf. [55,56,57]. Depending on the information source, there are between 1 and 6 million DHH people in Spain. In addition, minors cannot be registered as members of deaf people’s organisations, meaning it is even more difficult to estimate the exact number of DHH children in Spain. A total of 75 DHH children from 13 institutions (7 deaf people’s organisations and 6 schools with DHH students), living in 10 different Spanish cities, took part in the experiment.

The audiovisual material selected for the experimental study was kept short enough to avoid fatigue, bearing in mind the young age of participants, but at the same time was long enough to be audiovisually meaningful. To this end, all the videos contained a short story that retained their meaning outside the context of their episode or film. All the samples were between 1 and 3.5 min long. The final selection of video material took into account the presence of difficult vocabulary and the representation of practices shown in the descriptive study.

The subjects were divided into two groups, both of which watched the same number of videos (14) with either TV or ALT subtitling. No time limit was set for participants to complete the questionnaire and as a result, sessions lasted between 45 min and 2 h depending on the time participants needed, allowing time for a break if necessary. Two groups were formed in order to avoid watching the same video twice (once with TV and once with ALT subtitling) as that would imply previous knowledge of the content. Thus, subjects’ responses to a clip they had watched with TV subtitling were compared with responses from subjects who had watched the same clip with ALT subtitling, and vice versa.

Ad hoc questionnaires were created for each clip. As suggested by Alaminos and Castejón [58], and bearing in mind that DHH children might face difficulties or feel insecure when asked to write, all questions were multiple choice questions. For each question, only one correct answer was provided, along with two wrong answers and an “I am not sure” option, which is in line with recent research on SDH for children [17,59]. After watching each video, we stressed that the children were not supposed to guess as it was not a test, and encouraged them to mark the “I am not sure” option if they were in any doubt about the answer. We also offered to explain questions in oral or sign language and gave subjects the opportunity to explain their answer in oral or sign language to minimise the likelihood of their guessing the correct response. The questionnaires contained 25 questions on vocabulary and 16 on syntax. The number of questions for each variable depended on the content of the audiovisual product.

Before the experiment, all participants, assisted by their parents or tutors, filled out a demographic questionnaire similar to those used in previous research on SDH cf. [60]. This questionnaire included 19 questions regarding personal data (age, sex, etc.), hearing impairment data (hearing devices, degree of hearing loss, etc.), educational data (communication method, type of school, educational support, etc.), and TV viewing habits (TV stations, hours spent watching TV, frequency of use of subtitles, etc.), to help filter the data and analyse and discuss the results. These data were used as fixed factors in stepwise linear regression analysis to predict which of the subjects’ characteristics might indicate a better comprehension of the linguistic code of subtitles. Although the research had to address all the above-mentioned characteristics, the number of fixed factors might lower the power of the regression model, and the results and discussion derived from the regression model should therefore be considered with caution. To analyse the responses to the ad hoc questionnaires, we first analysed the distribution of correct, incorrect, and “I am not sure” responses in TV and ALT answers with the Kolmogorov–Smirnov test. Then, either parametric (t-Student) or non-parametric (Mann–Whitney *U* test) tests were used to analyse bilateral significance with IBM SPSS Statistics software, and unilateral significance with Minitab.

## 3. Results

A total of 6116 subtitles were analysed in the descriptive study, which identified 313 cases (5.1%) of difficult vocabulary in the spoken language. Of these 313 cases, vocabulary was adapted or omitted in 5.4% of the cases, compared with 94.6% in which it was transcribed literally into subtitles. The usual practice is therefore to use verbatim subtitles, in line with both the recommendations of the UNE Standard 153010 in Spain [54] and audience preferences. Regarding syntax, only 5% of the subtitles analysed had a structure other than subject-verb-object and 19.6% of the subtitles contained subordinate clauses. Verbatim transcription was also observed for syntax when technical limitations (maximum number of characters per line, for example) allowed it.

In the experimental study, 25 questions were asked about vocabulary (such as, “What is a “pagoda”?” or “What does the word “hypothesis” mean?”). As explained above, vocabulary was adapted to DHH children’s needs in two ways: by simplifying or omitting difficult vocabulary, and by using orthotypographic resources to enhance acquisition of new vocabulary, as shown in the figures below.

**Figure 1:**
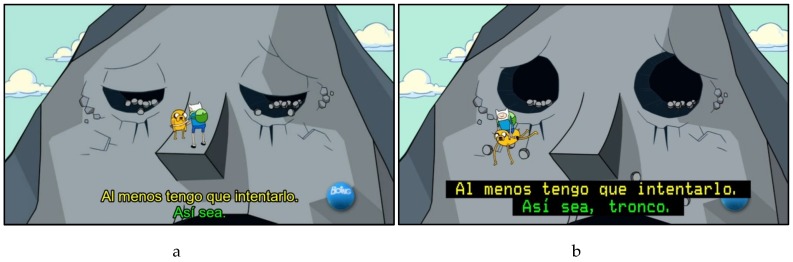
Verbatim TV subtitles (**b**) and omission of difficult vocabulary (*tronco*, colloquial word meaning *friend*) in ALT subtitling (**a**).

**Figure 2:**
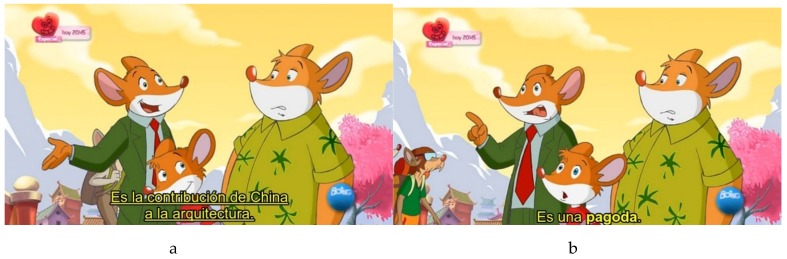
Both enhancement of new vocabulary (**b**) and its definition (**a**) in ALT subtitling to facilitate vocabulary acquisition.

Following recommendations in the literature [20,38,43], the decision of what strategy to take was based on the function of the vocabulary in its audiovisual context. Hence, when difficult vocabulary only reflected an idiosyncratic aspect of the character, it was modified or omitted (as shown in Figure 1b), but when the aim was to acquire difficult vocabulary, it was enhanced (as shown in Figure 2) and, if possible, left for longer on screen and repeated in later subtitles. Subtitle speed was reduced to 10 characters per second to allow longer time on screen whenever possible, that is, when there was no requirement for a change of subtitle resulting from a new shot or scene, or the intervention of another character. When repeated in subsequent subtitles, the enhanced word was also present in the soundtrack. Unlike the case of simple vocabulary, when difficult vocabulary appeared in the soundtrack, priority was given to repeating the word rather than including other elements; this sometimes led to further editing of the linguistic code (reformulation, condensation, or omission of information) to allow for repetition of difficult vocabulary.

Absolute frequencies of correct, incorrect, and “I am not sure” answers are shown in Table 2:

The linear regression reveals that, in general, four variables influence correct answers: communication method (more correct answers when children’s communication method is only oral, rather than bilingual or only signed), educational support outside school (more correct answers with no educational support outside school, such as speech therapy or reading and writing lessons), frequency of subtitling use on TV at home (the higher the frequency, the more correct answers), and educational support at school (more correct answers with educational support, such as review courses) (*R*^2^ = 0.229). These four variables influenced correct answers for ALT (*R*^2^ = 0.262), while only educational support outside school and communication method influenced correct answers for TV subtitling (*R*^2^ = 0.158). A model summary of the regressions for ALT and TV subtitling is shown in Table 3 and Table 4. As stated earlier, these results should be interpreted with caution due to the large number of fixed factors.

The Mann–Whitney *U* test reveals, in general, more correct answers (*p* = 0.0254) and also fewer incorrect answers (*p* = 0.012) with ALT subtitling than with TV subtitling, while there was no difference in the number of “I am not sure” answers (*p* = 0.418).

A total of 16 questions were asked to gauge syntax comprehension. Two main strategies were applied to adapt subtitles: using simple syntactic structures (subject-verb-object), and prioritising coordinate over subordinate clauses, whenever possible.

Absolute frequencies of correct, incorrect, and “I am not sure” answers are shown in Table 5:

The linear regression shows that, in general, the same four characteristics as in the vocabulary case influenced the number of correct answers (*R*^2^ = 0.244). When the data are filtered by type of subtitling, only the communication method influences both ALT (*R*^2^ = 0.137) and TV subtitling (*R*^2^ = 0.100). A model summary of the regressions for TV and ALT subtitling is shown in Table 6 and Table 7. As stated earlier, these results should be interpreted with caution due to the large number of fixed factors.

No significant differences are found between ALT and TV subtitling in the categories none, correct, incorrect, or “I am not sure” answers, although the trend is towards a higher number of correct answers in TV ($\bar{x}$ALT = 2.45, σALT = 1.98; $\bar{x}$TV = 2.48, σTV = 2.18).

## 4. Discussion

Generally speaking, fairly simple vocabulary is used in the dialogues in the corpus analysed in the descriptive study. Nevertheless, the few cases with difficult audible vocabulary tended to be transcribed verbatim into subtitles, following recommendations in the UNE Standard in Spain [54] but ignoring previous research such as that by Neves [14], Lorenzo [38], and Lorenzo and Pereira [43]. This practice might lead to difficulties in processing vocabulary, failure to understand the linguistic code, and, consequently, low motivation to read and poor language development.

Turning to the second variable, syntax, the corpus analysed in the descriptive study generally contained simple phrases and coordinate clauses, which seems to be in line with previous recommendations for research in this field. However, audiovisual products designed for young audiences would presumably bear in mind their cultural and linguistic background, and would therefore tend to have less complex structures and vocabulary.

In the experimental study, although omission or simplification of difficult vocabulary might entail a loss in pragmatic reward [61], on receiving audiovisual content, results for this variable prove empirically that some of the recommendations on simplification [14,38] and the use of orthotypographic resources [26] are useful for young DHH audiences. While previous research has focused on the lack of importance of language level in subtitling in visually rich audiovisual content (as in the case of audiovisual content designed for children and young audiences in which much information is transmitted through the visual channel or is made redundant by the oral channel and subtitles) [62,63] the results in this experiment show that language level does play a key role in the understanding of the linguistic code. Thus, verbatim subtitles, as recommended by the Spanish UNE Standard 153010 [54] and as preferred by deaf people’s organisations, do not imply better vocabulary comprehension. The results encourage us to press home the importance of avoiding verbatim subtitles in SDH for children, because edited (omitted or simplified) vocabulary increases comprehension, as some authors have previously suggested [41,64]. The decision to simplify or omit is never easy; recent guidelines, such as the BBC Subtitling Guidelines [65] advocate omissions, rather than simplification of vocabulary, to reduce sentence length and, thus, subtitle speed. Data in this study suggest that decisions on whether to adapt, omit, simplify, or enhance vocabulary have to be taken conscientiously, considering basic language issues, such as relevance and adequacy or function of the linguistic code in the audiovisual text. These results also bring to the fore a long discussed issue: the fact that SDH goes far beyond the pure transcription of oral into written text and that the subtitling practice needs to be carried out from a profoundly reflexive starting point to guarantee real accessibility. In other words, accessibility should be measured by how closely it satisfies the audience’s needs. Providing captions that do not meet the needs and the reading abilities of the audience they are intended for means the audiovisual product is not accessible, and to achieve accessibility, we must first start reflecting on the real and varying needs of the diverse groups that make up DHH audiences.

Regarding syntax, we cannot conclude from this study that the changes in the ALT subtitling lead to enhanced comprehension. Nevertheless, it must be borne in mind that current TV captioning for children already offers a simple syntactic structure in 95% of subtitles, and that subordinate clauses are absent in almost 80%. These results seem to be in line with recent SDH guidelines that recommend not simplifying sentences “unless the sentence construction is very difficult or sloppy” [65]. However, although our results were not statistically significant, we cannot ignore the debate between recommendations in previous research to simplify syntax when it does not involve distorting the narrative discourse [17,26,49,66,67], and the recommendations of current guidelines in favour of verbatim subtitles [54] and non-simplified syntax [65]. In addition, vocabulary has been shown to play a key role in the comprehension of syntactical structures [9,10,26]. For this reason, it seems advisable to edit the linguistic code so as to promote a balance between syntax and vocabulary levels. This type of editing should also provide more exposure time for difficult vocabulary and the gradual introduction of complex syntactic structures to allow the appropriate implementation of the key word strategy [9,10] so viewers are able to comprehend the linguistic code and audiovisual texts in their integrity.

## 5. Conclusions

In general, the way spoken language is transferred into SDH for children in Spain is fairly uniform. The linguistic code of audiovisual products designed for children in Spanish DTTV has simple syntax and vocabulary, and no specific effort is made to further simplify such language in the form of written subtitles to benefit language and content processing. This practice is in line with the official recommendations in Spain, but not with previous studies and suggestions drawn from research.

When the authors edited subtitles with the aim of improving subtitle processing, as seen in the experimental study, comprehension of vocabulary improved. Only significant results for edited subtitles (ALT) were found with adapted vocabulary, but not with adapted syntax. Be that as it may, audiovisual products have a complex multimodal and multisemiotic nature in which it seems difficult, and somewhat incoherent, to talk about only part of the linguistic code, as the audience will be processing the audiovisual text as a whole, with its images, its written text, and its acoustic channel, in the case of DHH audiences with residual hearing.

## Figures and Tables

**Table 1:** Changes made in the linguistic code to facilitate subtitle comprehension.

Changes in Vocabulary	Use of simple and denotative vocabulary that avoids abstract or figurative meaning when the intention of the audiovisual text is not to acquire such vocabulary.
	When difficult vocabulary is captioned, use of orthotypographic resources (bold and underlining) to enhance vocabulary, use of interphrasal redundancy, and reduction of subtitling speed, whenever possible.
Changes in Syntax	Use of simple phrasal structure (subject-verb-object structure) whenever possible.
	Avoidance of subordinate clauses and use of coordinate clauses, whenever possible.

**Table 2:** Frequency of answers for questions about vocabulary with alternative subtitles (ALT) and television subtitles (TV).

	ALT	TV
V-Correct	215	177
V-Incorrect	69	88
V-I Am not Sure	58	77

**Table 3:** Model summary of linear regression for vocabulary (TV).

Model Summary ^a^				
Model	*R*	*R* Square	Adjusted *R* Square	Standard Error of the Estimate
1	0.331 ^b^	0.110	0.097	2.42712
2	0.397 ^c^	0.158	0.134	2.37677

^a^ Type_subt = TV. ^b^ Predictors: (Constant), EDUCATION: external support. ^c^ Predictors: (Constant), EDUCATION: external support, IMPAIRMENT: communication.

**Table 4:** Model summary of linear regression for vocabulary (ALT).

Model Summary ^a^				
Model	*R*	*R* Square	Adjusted *R* Square	Standard Error of the Estimate
1	0.331 ^b^	0.109	0.097	2.77144
2	0.399 ^c^	0.159	0.136	2.71188
3	0.458 ^d^	0.210	0.177	2.64649
4	0.512 ^e^	0.262	0.220	2.57592

^a^ Type_subt = ALT. ^b^ Predictors: (Constant), IMPAIRMENT: communication. ^c^ Predictors: (Constant), IMPAIRMENT: communication, HABIT TV: subtitles. ^d^ Predictors: (Constant), IMPAIRMENT: communication, HABIT TV: subtitles EDUCATION: External support. ^e^ Predictors: (Constant), IMPAIRMENT: communication, HABIT TV: subtitles, EDUCATION: external support, EDUCATION: school support.

**Table 5:** Frequency of answers for questions on syntax with alternative subtitles (ALT) and television subtitles (TV).

	ALT	TV
S-Correct	135	139
S-Incorrect	90	80
S-I Am not Sure	64	71

**Table 6:** Model summary for linear regression syntax (TV).

Model Summary ^a^				
Model	*R*	*R* Square	Adjusted *R* Square	Standard Error of the Estimate
1	0.317 ^b^	0.100	0.084	2.08927

^a^ Type _subt = TV. ^b^ Predictors: (Constant), IMPAIRMENT: communication.

**Table 7:** Model summary for linear regression syntax (ALT).

Model Summary ^a^				
Model	*R*	*R* Square	Adjusted *R* Square	Standard Error of the Estimate
1	0.370 ^b^	0.137	0.121	1.85629

^a^ _subt = ALT. ^b^ Predictors: (Constant), IMPAIRMENT: communication.
